# Diagnostic Dilemma in a Case of Necrotizing Lymphadenitis With Macrophage Activation Syndrome

**DOI:** 10.7759/cureus.42267

**Published:** 2023-07-21

**Authors:** Akhila Arya P V, Md. Mashiul Alam, Andrew Bernhisel, Angela Degirolamo, Rex Huang

**Affiliations:** 1 Internal Medicine, Bridgeport Hospital, Bridgeport, USA; 2 Cardiovascular Disease, Mayo Clinic, Rochester, USA; 3 Pathology, Yale School of Medicine, Yale University, New Haven, USA; 4 Rheumatology, Yale University, New Haven, USA

**Keywords:** kikuchi-fujimoto systemic lupus erythematosus, sle initial diagnosis, kikuchi disease, c4d staining, systemic lupus erythematosus with macrophage activation syndrome

## Abstract

Necrotizing lymphadenitis is a histological diagnosis that can arise from various conditions, including lupus lymphadenitis (LL), Kikuchi disease (KD), and infectious causes. Distinguishing between Kikuchi disease and lupus lymphadenitis can be challenging in clinical practice. In this report, we present the clinical scenario of a young female patient with lymphadenopathy and elucidate the process through which we ultimately arrived at a diagnosis of systemic lupus erythematosus (SLE) with macrophage activation syndrome. This case underscores the significance of recognizing Kikuchi disease as a condition that can mimic lupus and sheds light on the distinguishing features of necrotizing lymphadenitis, with a particular focus on Kikuchi disease and lupus lymphadenitis.

## Introduction

Necrotizing lymphadenitis is a histological diagnosis that can result from a wide variety of conditions - lupus lymphadenitis (LL), histiocytic necrotizing lymphadenitis also known as Kikuchi disease, and infectious causes (like herpes simplex virus {HSV} and Epstein-Barr virus {EBV}) [1]. Lymphadenitis associated with EBV and HSV often exhibits distinct serologic, morphologic, and immunohistochemistry features, facilitating their differentiation from other entities. Conversely, distinguishing between Kikuchi disease and lupus lymphadenitis can be challenging due to overlapping clinical and pathological characteristics. Therefore, a comprehensive approach encompassing clinical presentation, serological investigations, histopathology, and immunohistochemistry is crucial for accurate diagnosis. C4d staining on biopsy specimens has emerged as a novel method for differentiating Kikuchi disease from systemic lupus erythematosus (SLE) [2]. Additionally, it is important to note that macrophage activation syndrome can be present in all potential causes of necrotizing lymphadenitis and necessitates aggressive management.

## Case presentation

A 24-year-old Hispanic female with no significant medical history presented with a two-week history of fever and myalgias. Upon initial examination, she had a fever of 103°F accompanied by tachycardia and hypotension. Physical examination revealed bilateral mobile cervical lymphadenopathy that was non-tender. No rashes or organomegaly were observed during the examination. Cardiac and lung auscultation did not reveal any abnormalities.

Labs at admission and further workup are tabulated below. The provisional diagnosis upon admission was sepsis of an unknown source, and the patient was promptly started on antibiotics. However, initial blood and urine cultures did not show any growth. Furthermore, extensive infectious workups conducted yielded negative results (Tables 1, 2).

**Table 1:** Basic and autoimmune laboratory parameters of patient. WBC: white blood cells; BUN: blood urea nitrogen; CRP: C-reactive protein; LDH: lactate dehydrogenase; PR3: proteinase 3; MPO: myeloperoxidase

Parameter		Patient’s value	Reference range
Basic labs	Hemoglobin	12.4 g/dL	11.7-15.5 g/dL
	Platelets	173×1000/µL	150-420×1000/µL
	WBC	3.2×1000/µL	4.0-11.0×1000/µL
	Neutrophils	82.6%	39.0-72.0%
	Lymphocytes	6.9%	17.0-50.0%
	Sodium	133 mmol/L	136-144 mmol/L
	Potassium	3.5 mmol/L	3.3-5.3 mmol/L
	BUN	11 mg/dL	6-20 mg/dL
	Creatinine	0.81 mg/dL	0.40-1.30 mg/dL
	Total protein	7.5 g/dL	6.6-8.7 g/dL
	Albumin	4.2 g/dL	3.6-4.9 g/dL
	Total bilirubin	0.4 mg/dL	1.2 mg/dL
	Alanine aminotransferase	18 U/L	10-35 U/L
	Aspartate aminotransferase	48 U/L	10-35 U/L
Autoimmune labs	Antinuclear antibodies (ANA)	Positive, 1:160, speckled	<1:40
	dsDNA antibody	<12.3 IU/mL	<30 IU/mL
	Sm antibody	4.0	<1.0 NEG AI
	Sm/RNP antibody	1.6	<1.0 NEG AI
	SS-A, SS-B	<1.0	<1.0 NEG AI
	C3	62 mg/dL	90-180 mg/dL
	C4	22 mg/dL	10-40 mg/dL
	PR3, MPO antibody	<1.0	<1.0 NEG AI
	Liver kidney microsomal and smooth muscle antibody	<2.0 Units	<20.0 Units
	Ferritin	22,665 ng/mL	13-150 ng/mL
	Triglycerides	323 mg/dL	<150 mg/dL
	LDH	2052 U/L	122-241 U/L
	CRP	127.4 mg/L	>10.0 consistent with infection/inflammation
	Fibrinogen	360 mg/dL	187-446 mg/dL
	Interleukin 2 receptor	8637 pg/mL	532-1891 pg/mL

**Table 2:** Infectious disease workup. PCR: polymerase chain reaction; RPR: rapid plasma reagin; HIV: human immunodeficiency virus; EBV: Epstein-Barr virus; CMV: cytomegalovirus; HSV: herpes simplex virus; RSV: respiratory syncytial virus; VCA: viral-capsid antigen; HBsAg: hepatitis B surface antigen

Parameter	Result
Blood cultures	No growth after 5 days of incubation
Urine culture	No growth after 5 days of incubation
Malaria smear	Negative for parasites
Babesia smear	Negative for parasites
Anaplasma, Ehrlichia PCR	Not detected
Francisella tularensis, Lyme antibody	Negative
RPR with reflex titer	Negative
Hepatitis B (HBsAg, IgM core antibody)	Non-reactive
Hepatitis C antibody	Non-reactive
HIV 1 and 2 antibody	Non-reactive
EBV VCA IgM	Negative
Rubella IgM/IgG	Negative
CMV quantitative PCR	Not detected
HSV 1, 2 PCR	Not detected
SARS-CoV, influenza, RSV	Negative
Parvovirus IgM	Negative
QuantiFERON TB	Negative
Fungitell (1-3) B D glucan assay	Negative
Histoplasma antigen urine	Negative

Subsequently, the patient developed a sore throat, and a computed tomography (CT) scan of the neck, chest, abdomen, and pelvis was performed. The CT scan revealed extensive lymphadenopathy in the hilar, mediastinal, and axillary regions, as well as bilateral mild to moderate pleural effusion. To further investigate the condition, both a bone marrow biopsy and a cervical lymph node biopsy were conducted.

**Figure 1:**
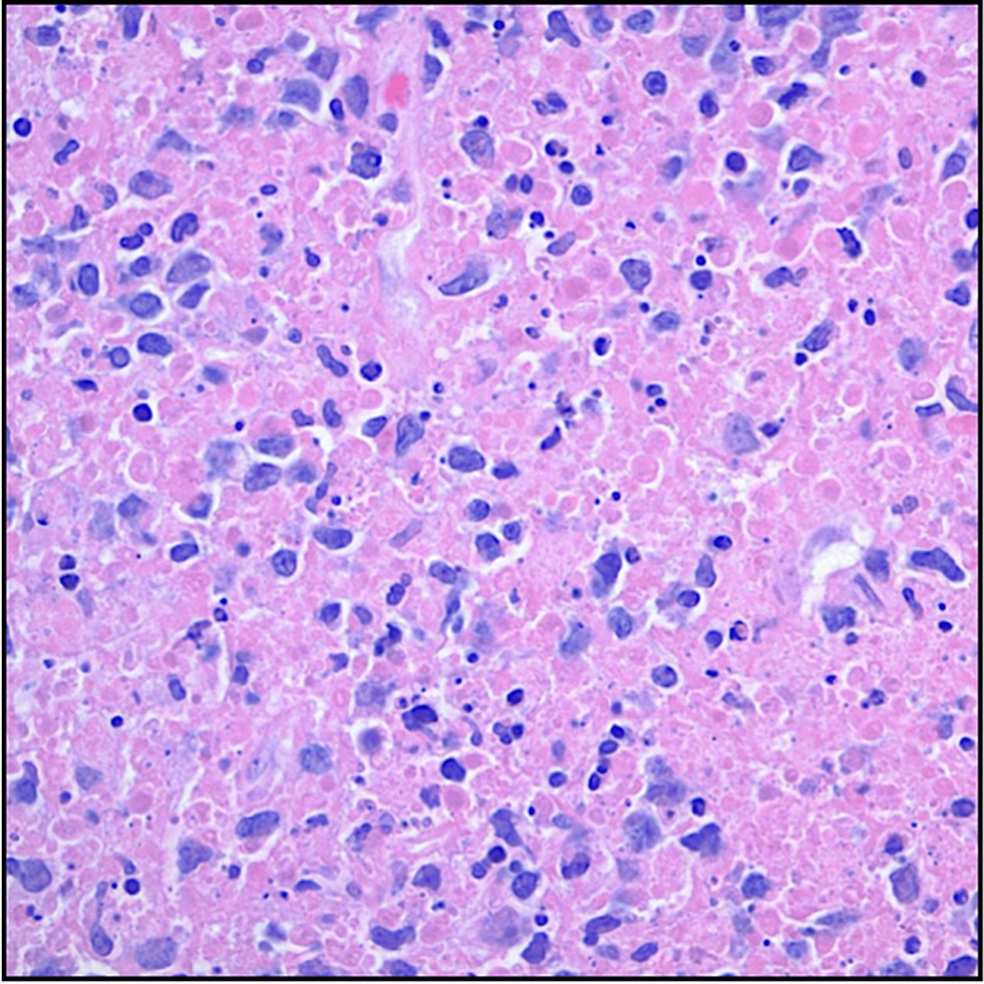
Markedly necrotic lymphoid tissue and karyorrhectic debris without accompanying neutrophils on lymph node biopsy (H&E, 200×).

The bone marrow biopsy demonstrated normal development of all three blood cell lineages without any evidence of lymphoma. The lymph node biopsy, on the other hand, revealed areas of necrosis with the presence of karyorrhectic debris and apoptosis. Neutrophils were not observed, but scattered histiocytes and plasma cells were present (Figure 1). The lymph node cells tested positive for CD123 highlighting the presence of plasmacytoid dendritic cells (abundant in Kikuchi disease). Based on these findings, the pathology was suggestive of either Kikuchi disease or lupus lymphadenitis.

**Figure 2:**
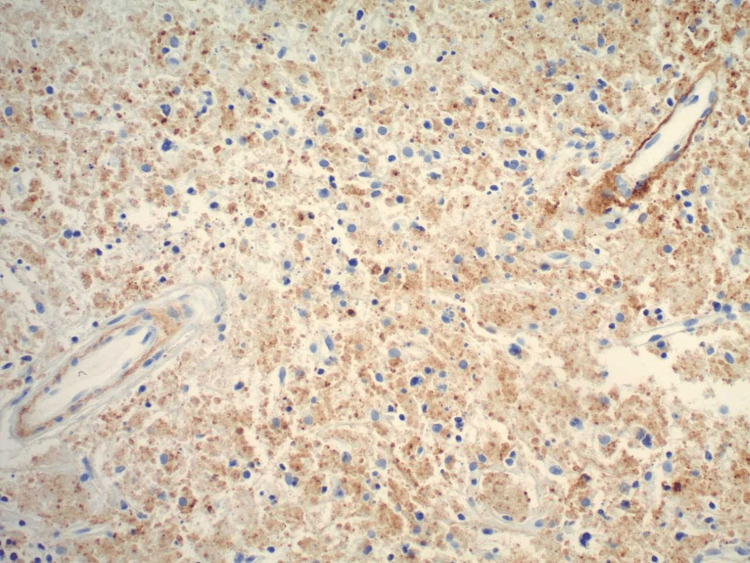
C4d-positive staining around vessels favoring diagnosis of SLE over Kikuchi disease (100×). SLE: systemic lupus erythematosus

To differentiate between the two possibilities, C4d staining of the biopsy specimen was performed (vide infra). The staining revealed positive results in the necrotic areas and around the blood vessels, providing evidence in favor of lupus lymphadenitis over Kikuchi disease (Figure 2).

The patient's clinical course was complicated by worsening pancytopenia, transaminitis, oral ulcers, and persistent high-grade fevers. This raised suspicion of the development of hemophagocytic lymphohistiocytosis (HLH) or macrophage activation syndrome (MAS). Laboratory investigations revealed hyperferritinemia, hypertriglyceridemia, elevated lactate dehydrogenase (LDH) levels, hypocomplementemia, elevated soluble IL-2 receptors, and elevated C-reactive protein (CRP) levels (Table 1). H score, a scoring system used to assess the probability of hemophagocytic lymphohistiocytosis (HLH) or macrophage activation syndrome (MAS), was calculated to be 196. This score suggests an 80-88% probability of HLH/MAS. In addition, the patient fulfilled five points on the HLH 2004 criteria, further supporting the possibility of HLH/MAS (Table 3).

**Table 3:** H score calculation in our patient. AST: aspartate aminotransferase; HIV: human immunodeficiency virus

Parameter	Result	Score
Known underlying immunosuppression (HIV positive or receiving long‐term immunosuppressive therapy (i.e., glucocorticoids, cyclosporine, azathioprine)	No	0
Temperature (°F)	>102.9	+49
Organomegaly hepatomegaly and/or splenomegaly)	No	0
Number of cytopenias	3 lineages	+34
Ferritin (ng/mL)	>6000	+50
Triglyceride (mg/dL)	132.7-354	+44
AST (U/L)	≥30	+19
Hemophagocytosis features on bone marrow aspirate	No	0
Total score		196

Additional laboratory findings included an elevated antinuclear antibody (ANA) titer of 1:160 with a speckled pattern. Anti-Sm and anti-Sm RNP antibodies were also elevated (Table 1). Based on the 2019 European League Against Rheumatism (EULAR)/American College of Rheumatology (ACR) criteria for systemic lupus erythematosus (SLE), a probable diagnosis of SLE was made.

These findings collectively indicate a probable diagnosis of SLE according to the 2019 EULAR/ACR criteria, along with the presence of features suggestive of HLH/MAS based on the H score and HLH 2004 criteria. It took us about 10-12 days to reach this diagnosis.

Consequently, the patient received treatment with 1 mg/kg methylprednisone IV twice a day, which resulted in substantial clinical improvement as well as normalization of laboratory parameters. As her condition continued to improve, she was discharged from the hospital with a gradually tapering dose of steroids and prescribed hydroxychloroquine. Additionally, azathioprine was initiated as a steroid-sparing therapy to minimize long-term steroid usage.

During follow-up visits, the patient demonstrated complete resolution of both clinical symptoms and abnormal laboratory findings. Her functional status returned to baseline, indicating a restoration of her overall health and well-being. This positive outcome highlights the effectiveness of the treatment regimen, consisting of high-dose steroids, hydroxychloroquine, and the addition of azathioprine as a maintenance therapy to reduce the reliance on long-term steroid use.

## Discussion

The main differentials for non-granulomatous necrotizing lymphadenitis include HSV and EBV infections, lupus lymphadenitis, and Kikuchi's disease. While it is relatively straightforward to rule out the first four causes due to their distinct morphological and immunohistochemical features, differentiating between lupus lymphadenitis and Kikuchi disease histologically can be challenging. Typically, this differentiation necessitates robust clinical and serological correlation (vide infra) [3].

HSV lymphadenitis is rare, characterized by the presence of neutrophils and potential nuclear inclusions, and immunohistochemistry (IHC) for HSV can aid in the diagnosis [4]. In this case, HSV infection was excluded based on negative IHC and serological results, while EBV serologies also yielded negative findings. Consequently, the differential diagnoses were narrowed down to lupus lymphadenitis or Kikuchi's disease.

Histiocytic necrotizing lymphadenitis, commonly known as Kikuchi disease or Kikuchi-Fujimoto disease, is a benign condition characterized by cervical lymphadenopathy. It was first described by Kikuchi in 1972 in Japan [1]. Although the exact cause is unknown, both infectious and autoimmune factors have been proposed [5]. The epidemiology of Kikuchi disease is like SLE, with a higher prevalence in young females. Due to the rarity of the disease, exact incidence is unknown. The underlying pathogenesis is believed to involve defective cellular immunity, leading to apoptotic cell death [5]. Morphologically, this is reflected as nuclear chromatin condensation, fragmentation of the nuclear membrane with intact organelles, and histiocytes engulfing the resulting karyorrhectic debris. The clinical presentation of Kikuchi disease commonly includes lymphadenopathy, fever, rash, arthritis, fatigue, and hepatosplenomegaly [6]. Serological studies such as antinuclear antibodies (ANA) and rheumatoid factors are typically negative unless the patient has underlying SLE that develops subsequently. Kikuchi disease and SLE can occur simultaneously, as in the presented case, or Kikuchi disease may precede or follow the development of SLE. This has led to the argument that Kikuchi disease falls within the spectrum of SLE [3].

According to Kuo, Kikuchi disease can be categorized into three pathological stages [7]. In the early proliferative phase, there is follicular hyperplasia, expansion of the paracortex by lymphocytes, T/B cell blasts, plasmacytoid and myeloid dendritic cells, as well as histiocytes containing numerous apoptotic or karyorrhectic nuclear debris. It is important to note that nodal architecture should be preserved, and the infiltrating lymphocytes should display a polyclonal pattern. The presence of a large proportion of blasts raises suspicions of lymphoma, EBV, or HSV as possible etiologies.

During the necrotizing phase, histiocytes with crescentic nuclei and engulfed debris become predominant. These histiocytes typically stain positive for CD68, and CD8+ T cells are also observed. The absence of neutrophils within the necrotic areas, the presence of vasculitis surrounding the necrosis, and the identification of clusters of basophilic hematoxylin bodies within the lymph node are suggestive of systemic lupus erythematosus (SLE). In 2021, Yu et al. proposed that immunohistochemical staining for C4d, a marker of antibody-mediated complement activation, can be useful in differentiating between Kikuchi disease and SLE. The presence of C4d staining favors the diagnosis of SLE over Kikuchi disease [2]. Additionally, CD68 and CD123, which are markers of plasmacytoid dendritic cells, serve as surrogate markers for the diagnosis of Kikuchi disease [8]. Additionally, CD68 and CD123, which are markers of plasmacytoid dendritic cells, serve as surrogate markers for the diagnosis of Kikuchi disease.

The typical course of Kikuchi disease is self-limiting, with resolution occurring within a few months. However, in severe cases, treatment options such as glucocorticoids and intravenous immunoglobulin have been described. Complications of Kikuchi disease can include recurrences, progression to SLE, and the development of macrophage activation syndrome (MAS).

In the present case, the initial presentation of prolonged fever and generalized lymphadenopathy, which is not very typical of SLE, along with the pathological findings of necrosis without neutrophils, raised suspicion for Kikuchi disease. However, as serological markers later returned positive for SLE and the patient met the diagnostic criteria for SLE, the diagnosis was revised. Coexistence of both Kikuchi disease and SLE has been well-documented, suggesting the possibility of their concurrent presence in this case [9]. Additionally, the worsening cytopenia observed during the illness prompted consideration of the development of macrophage activation syndrome.

MAS and HLH are related terms that share some overlap in the etiologies that can cause them. Hemophagocytic lymphohistiocytosis (HLH) is a potentially life-threatening hematological syndrome characterized by dysregulation of the immune response, which can occur in response to infections, autoimmune disorders, or malignancies, leading to multiorgan dysfunction. Macrophage activation syndrome (MAS) is a term often used specifically for HLH associated with rheumatological conditions. The incidence of HLH in Kikuchi disease is limited, but a study by Ahn et al. in 2019 reported that 30% of Kikuchi disease patients had MAS at presentation [10]. The occurrence of MAS in SLE ranges from 0.9% to 4.6% [11]. A review article by Aziz et al. in 2021 found that SLE flare and high SLE Disease Activity Index (SLEDAI) scores were predictors of MAS development [12]. There are no single criteria for diagnosis, some commonly used ones are the H score which uses 10 variables, or a score of ≥5 in HLH 2004 criteria [13-14]. Our patient had an H score of 203, suggesting an 80-93% probability of HLH, and scored 5 in the HLH 2004 criteria (which includes fever, cytopenias, hypertriglyceridemia, elevated ferritin and elevated soluble IL-2 receptor as criteria) in the typical clinical context.

## Conclusions

Necrotizing lymphadenitis due to SLE can closely resemble that of Kikuchi disease. MAS can be an associated complication with high risk for mortality, clouding the diagnosis. There should be high clinical suspicion and rheumatologic workup to rule out SLE in such cases. C4d can be a potential immunological marker to aid in differentiating the two.
